# Comparison of Perceived and Technical Healthcare Quality in Primary Health Facilities: Implications for a Sustainable National Health Insurance Scheme in Ghana

**DOI:** 10.1371/journal.pone.0140109

**Published:** 2015-10-14

**Authors:** Robert Kaba Alhassan, Stephen Opoku Duku, Wendy Janssens, Edward Nketiah-Amponsah, Nicole Spieker, Paul van Ostenberg, Daniel Kojo Arhinful, Menno Pradhan, Tobias F. Rinke de Wit

**Affiliations:** 1 Amsterdam Institute for Global Health and Development (AIGHD), Amsterdam, Netherlands; 2 Department of Epidemiology, Noguchi Memorial Institute for Medical Research (NMIMR), University of Ghana, Legon, Accra, Ghana; 3 Department of Economics, University of Ghana, Legon, Accra, Ghana; 4 PharmAccess Foundation, Amsterdam, Netherlands; 5 Department of Development Economics, Vrije University (VU), Amsterdam, Netherlands; 6 Joint Commission International (JCI), Chicago, United States of America; 7 University of Amsterdam (UvA), Amsterdam, Netherlands; Kenya Medical Research Institute - Wellcome Trust Research Programme, KENYA

## Abstract

**Background:**

Quality care in health facilities is critical for a sustainable health insurance system because of its influence on clients’ decisions to participate in health insurance and utilize health services. Exploration of the different dimensions of healthcare quality and their associations will help determine more effective quality improvement interventions and health insurance sustainability strategies, especially in resource constrained countries in Africa where universal access to good quality care remains a challenge.

**Purpose:**

To examine the differences in perceptions of clients and health staff on quality healthcare and determine if these perceptions are associated with technical quality proxies in health facilities. Implications of the findings for a sustainable National Health Insurance Scheme (NHIS) in Ghana are also discussed.

**Methods:**

This is a cross-sectional study in two southern regions in Ghana involving 64 primary health facilities: 1,903 households and 324 health staff. Data collection lasted from March to June, 2012. A Wilcoxon-Mann-Whitney test was performed to determine differences in client and health staff perceptions of quality healthcare. Spearman’s rank correlation test was used to ascertain associations between perceived and technical quality care proxies in health facilities, and ordered logistic regression employed to predict the determinants of client and staff-perceived quality healthcare.

**Results:**

Negative association was found between technical quality and client-perceived quality care (coef. = -0.0991, p<0.0001). Significant staff-client perception differences were found in all healthcare quality proxies, suggesting some level of unbalanced commitment to quality improvement and potential information asymmetry between clients and service providers. Overall, the findings suggest that increased efforts towards technical quality care alone will not necessarily translate into better client-perceived quality care and willingness to utilize health services in NHIS-accredited health facilities.

**Conclusion:**

There is the need to intensify client education and balanced commitment to technical and perceived quality improvement efforts. This will help enhance client confidence in Ghana’s healthcare system, stimulate active participation in the national health insurance, increase healthcare utilization and ultimately improve public health outcomes.

## Introduction

Low adherence to quality care practices in health facilities remains one of the reasons why many low and middle income countries (LMICs) in Africa are unable to meet the targets of the health-related Millennium Development Goals (MDGs) [1]. Although quality in healthcare delivery has been examined from different perspectives in the past [2,3,4] it was not until the last two decades that the topic gained prominence as a means to enhance effectiveness and accountability in health systems, especially in Africa [5]. Adherence to quality healthcare practices remains low in many LMICs largely due to financial, logistical and human resource constraints. Moreover, available community resources and potentials are often inadequately harnessed to support central governments’ efforts.

Quality healthcare is often categorized into technical and perceived [2], with the former referring to structured processes and professionally defined practices and protocols of care while the latter focuses on perceptions, experiences and satisfaction with the service delivery processes [2,6]. The Institute of Medicine (IOM) in the United States of America (USA) proposed six attributes of quality healthcare indicating that healthcare should be client-centered, timely, effective, efficient, safe and equitable [7].

Within the Ghanaian context, quality in healthcare has been investigated from different dimensions in recent past [8–11] and continues to draw attention of researchers and the Institutional Care Division (ICD) of the Ghana Health Service (GHS) [12] because of the increasing relevance of quality care in health insurance sustainability and population health outcomes [13]. The introduction of the National Health Insurance Scheme (NHIS) in Ghana barely a decade ago particularly pose substantial challenge to healthcare facilities to maintain acceptable healthcare quality standards. Moreover, empirical evidence of low patient satisfaction with health service quality [8–11] coupled with minimal gains in health outcome indicators have put healthcare quality at the centre of Ghana’s healthcare system.

Many scientific studies on quality care in Ghana either investigate quality healthcare solely from the medical technical or client-perceived perspectives without comparing the two dimensions, particularly in the context of accredited health facilities. For instance, studies by Turkson [11] and Atinga et al [8–10] on quality healthcare in Ghana did not compare perceptions of patients and health staff with patient safety and risk reduction efforts in the pertinent health facilities. Other previous studies on quality healthcare in Ghana [14–16] and some African countries [5,17,18,19] largely focused on client perception/satisfaction variables to “measure” quality healthcare.

While acknowledging the importance of client-perceived quality healthcare in quality improvement plans [17,19,20], it is important to recognize that mainly relying on this quality dimension might not adequately define the whole concept of quality healthcare and should not be used alone as proxy for overall quality, safety and effectiveness of a healthcare system [21]. Likewise, relying mainly on technical quality assessment outcomes without taking into account the experiences and views of clients might not enhance quality from the perspective of clients which is needed to increase health service utilization and health insurance uptake and coverage. Verifying perceived quality care with technical healthcare quality standards offers researchers and health policy makers the opportunity to identify expectations of clients and healthcare providers, and determine more wholistic quality improvement interventions.

Even though there are limitations associated with using client satisfaction surveys to “measure” quality healthcare, adequate triangulation of perceived and technical quality assessment methods could prove useful in determination of quality care situations in healthcare facilities. For instance, there are objectivity and reliability concerns when researchers exclusively depend on client perceptions to ascertain quality of healthcare in health facilities because of potential client intimidation arising from interviews conducted within health facility environs [5,6,21,22]. The tendency for clients to respond favorably to questions on quality healthcare dimensions could be high but not necessarily reflect their experiences and judgment of the quality situation. Robyn et al [23] found that even though insured clients in Burkina Faso rated quality healthcare dimensions high, these clients actually received lesser technical quality care in terms of physical examinations and adherence to other standard protocols by health personnel.

Favorable responses by clients in many instances could be attributed to the fact that most clients, especially in rural Africa, have limited knowledge of what constitutes quality healthcare or they simply do not have enough health facility alternatives to compare quality standards. In addition, perceptions of clients on quality healthcare are often influenced by attributes such as gender, age, cultural orientation, religion, geographic location (rural or urban) and income levels [5,6,8–11,15]. These attributes if not appropriately adjusted for could skew responses and possibly misinform researchers’ conclusions. In view of these limitations, a comparison of client/staff- perceived quality care with technical quality proxies (also referred to as patient safety and risk status in this paper) could offer a better understanding of the quality situation in pertinent healthcare facilities.

This study is motivated by the existing limitations in the literature especially on Ghana where the introduction of the National Health Insurance Scheme (NHIS) and its accreditation system has increased the need for a multi-faceted approach to healthcare quality improvement, especially in accredited primary health facilities which constitute over 70% of the 3,575 health facilities accredited by the NHIA as at 2012. Understanding views of clients and health providers on quality care and comparing these views with the technical quality care situation in the particular health facilities will offer policy makers and health managers the opportunity to address existing gaps in the service delivery process and promote client trust in the healthcare system and the NHIS. Applying triangulated approaches in investigating healthcare quality, as demonstrated in Dalinjong and Laar [13], De Man et al [24], Borgermans et al [25], Ackermann et al [26] and Drain [27], will help health managers to do adequate introspection and at the same time understand clients’ expectations which are needed to design appropriate client-centered healthcare quality improvement interventions.

This paper sought to ascertain the perceptions of clients and health staff on quality healthcare services in accredited primary health facilities and how these perceptions correlate with patient safety and risk status (technical quality) in these facilities. The expectation is that a comprehensive exploration of healthcare quality will help attain healthcare systems that are client-centered yet timely, effective, efficient, safe and equitable in resource poor settings in Africa.

The following research questions are addressed in the paper: i) Are there differences in perceptions of clients and health staff on quality health service delivery in accredited health facilities? If so what are these differences? ii) Do client and provider perceptions of service quality correlate with an objective assessment of patient safety and risk status (technical quality) in sampled NHIS-accredited facilities in Ghana?

## Materials and Methods

### Study design and data collection

This paper reports on findings of a baseline study which is part of a Randomized Control Trial (RCT) project initiated in 2011 aimed at improving (re)enrolment rates in Ghana’s NHIS through client-centered quality healthcare (see Alhassan et al, [28]). The baseline study design included household and health facility level surveys conducted in the catchment area of 64 sampled NHIS-accredited clinics/health centres. In addition, clinic staff interviews were conducted alongside patient safety and risk status assessment in these same health facilities. The patient safety and risk reduction indicators were used as proxies for technical quality. The term “technical quality” is thus used to represent findings of the patient safety and risk reduction assessments.

### Study sites and context

There are over 5, 000 private and public healthcare facilities serving an estimated population of 26.9 million people in 10 administrative regions in Ghana. Out of this number, 3,575 have been accredited (licensed) to render services to NHIS subscribers [29].

This study was conducted in the Greater Accra and Western regions located in the southern part of Ghana. The Greater Accra region (GAR) is predominantly urban and cosmopolitan with close to 4 million people and 416 NHIS-accredited healthcare facilities. The Western region (WR) is largely rural with a population of a little over 2 million people and 438 NHIS-accredited health facilities [29]. Out of the estimated 8.9 million active membership in Ghana’s NHIS in 2012, 13.5% and 10.1% of them were resident in GAR and WR respectively [29]. There are 144 NHIA district offices; out of this number, 10 are in GAR and 15 in WR. The NHIA district offices do not represent administrative districts since not all administrative districts necessarily have an NHIA district office.

### Sampling procedure

The sampling procedure was a mixed-methods approach using probability and non-probability sampling techniques at the district, health facility, health staff and household levels.

#### Sampling NHIS districts

A total of 16 NHIS district offices, 8 in each region, were purposively sampled for the study and used as proxies for administrative districts. All the districts offices eligible for selection into the study had one NHIS district office serving the population. Principal component analysis (PCA) was used to select the NHIS districts and health facilities to ensure comparability. The PCA was used to generate scores for the districts offices and catchment area using (i) the district population, (ii) enrolment rate, (iii) number of NHIS-accredited facilities per 1,000 population and (iv)number of non-accredited facilities per 1,000 population. Based on these parameters, 8 NHIS district offices with same or almost same PCA scores were purposively sampled from each region for inclusion in the study.

#### Sampling health facilities

At the district level, NHIS accreditation data on all primary health facilities in the 16 sampled districts was used to generate PCA scores. Next, in each district 4 health facilities with the same or almost the same scores were sampled to ensure that the facilities were comparable. Per this criterion, a total of 64 health facilities (32 in each region) were sampled. The 32 facilities from each region represented approximately 28% of accredited primary health facilities in the Western and Greater Accra regions as at 2012. Only NHIS-accredited health facilities were purposively sampled for the study because of the primary focus on healthcare quality in the context of the NHIS.

#### Sampling households

The catchment area of the sampled health facilities was chosen as the preferred option for sampling the households. Thereafter, between 3 and 5 enumeration areas (EAs) were identified within the catchment area of each health facility with the help of EA maps obtained from the Ghana Statistical Service (GSS). The sampling of households was done within a 10km radius of the 64 sampled health facilities to ensure that clients’ responses were relevant to the quality of services rendered in these pertinent health facilities. During the interviews, respondents were asked if they have accessed their nearest health facility for health services in the past six months. This formed part of the criteria for proceeding to ask questions related to their experiences with the quality of health services. All residential buildings within the selected EAs were listed followed by a random sampling of 30 residential buildings from the selected EAs. The random sampling of these residential buildings was such that the number selected from each EA has probability proportional to the number of buildings listed in that EA. Per this criterion 30 households were randomly sampled, one from each of the selected residential buildings. The number of households within each residential building was identified based on the study’s definition of household. Household is operationally defined to consist of a person or group related or unrelated, who live together in the same housing unit, and share the same housekeeping and cooking arrangements. The housing unit acknowledges one adult male or female as the head of the household, and are considered as one unit.

#### Sampling health staff

At the health facility level, clinical (n = 272) and support staff (n = 52) with at least 6 months working experience were randomly sampled from the 64 facilities. To prevent potential skewed responses, at most one respondent from each available professional category was randomly sampled and interviewed. The categories of clinical health personnel involved in the study include: medical doctors, medical assistants, professional nurses, nurse-assistants, pharmacist, pharmacist-assistants, midwives, laboratory technologists and laboratory technicians. The support staff include health service administrators, accounting staff, secretaries, receptionists, NHIS contact persons and medical records officers.

### Instruments of data collection

Three main instruments were used for the primary data collection, namely the *SafeCare Essentials* tool to “measure” technical quality of care, a health facility staff questionnaire to ascertain staff perceptions of quality, and a household questionnaire to determine client perceptions of the quality of health care. The SafeCare Essentials tool used to assess patient safety and risk reduction efforts in the selected facilities is provided by the SafeCare Initiative, a collaboration of the PharmAccess Foundation, the Council for Health Services Accreditation of Southern Africa (COHSASA), and the Joint Commission International (JCI). The tool is designed to identify the capability of a facility to move slowly or more rapidly towards higher levels of clinical quality and safer patient care according to staff efforts [30].

The SafeCare Essentials tool comprised of 41 assessment criteria categorized into five risk areas. The five primary risk areas are: leadership and accountability (7 criteria); competent and capable workforce (7 criteria); safe environment for staff and patients (10 criteria); clinical care of patients (10 criteria), and improvement of quality and safety (7 criteria). Each assessment criteria is scored on a four-point scale (0–3) called “Levels of Effort”. High levels depict better efforts by staff of pertinent health facility towards enhancing patient safety and reducing risk (i.e proxy technical quality care).

During implementation of the SafeCare Essentials tool, a health facility is scored Zero (0) for a particular quality criterion if the desired quality improvement activity is absent or there is mostly ad hoc activity related to risk reduction. One (1) is scored when the structure of more uniform risk-reduction activity begins to emerge in the pertinent health facility. Two (2) is scored when there are processes in place for consistent and effective risk-reduction in the health facility. Three (3) is scored when there are data to confirm successful risk-reduction strategies and continuous improvement.

Personal digital assistant (PDA) devices were used by two trained research assistants to do double scoring per healthcare facility. The two research assistants later reconciled scores after every assessment. The assessments were done objectively using a combination of direct observations, interviews with health managers, inspection and verification from administrative records (excluding patient medical records).

The SafeCare Essentials tool is deemed appropriate for the Ghanaian and African context because it has been used in over 2,000 health facilities in Ghana, Nigeria, South Africa, Kenya, Mozambique and Namibia prior to its adoption in this study [30].

For the purposes of our analysis, mean percentage (%) scores were computed for each of the sampled health facilities based on their scores on the 41 assessment criteria. For every health facility, the mean % scores were computed by summing all applicable criteria scores (0–3) under each risk area divided by the total expected score per risk area and multiplied by 100. High mean % scores thus depict better levels of effort towards patient safety and quality by staff and vice versa. To attain the overall “technical quality” score per health facility, the mean scores in the five primary risk areas were summed. In view of this scoring design, the risk assessment scores on each of the 64 sampled health facilities were used as proxy indicators for technical quality care.

The household and health facility staff questionnaires explored respondents’ socio-demographic characteristics, employment status of household heads, professional category, insurance enrolment status and perceptions on quality of healthcare services in the nearest NHIS-accredited health facility.

Household heads and health staff were asked triangulated questions on perceptions of healthcare quality by ranking their levels of satisfaction with service quality in the following areas: avenues/places for complaint lodging; process of lodging complaint; compassion and supportiveness of health staff; respectfulness of health staff; equal treatment of insured and uninsured patients; adequacy of consulting rooms and medical equipment; access to all prescribed drugs at the facility; overall satisfaction with health services provided by facility; information provided by facility; sufficiency of medical staff, and overall waiting time at the facility. The household questions on the various healthcare quality proxies were on a 5 point Likert scale from 1 = “Very dissatisfactory” to 5 = “Very satisfactory” while the health facility staff questions were on a 4 point Likert scale from 1 = “Very dissatisfactory” to 4 = “Very satisfactory”.

Different measurement scales were used because the surveys for clients and staff were conducted separately though concurrently. Though there is no explicit scientific opinion on this approach, intuitively it was meant to promote reliability in responses under the circumstances. The scale reliability for the 12 Likert scale items was checked and Cronbach’s alpha found to be 0.86 and 0.70 for household and health staff responses respectively which are within the 0.70–1.00 rule of thumb [31,32].

The data collection tools were piloted in one conveniently sampled district in the Greater Accra region. The aim of the pilot was to help enhance the scientific rigor, feasibility and value of the full-scale study.

### Ethical considerations

Ethical clearance for the study was obtained from the Ghana Health Service (GHS) Ethical Review Committee (ERC) [clearance numbers: GHS-ERC: 18/5/11and GHS-ERC 08/5/11]. Informed consent was also obtained from individual respondents in the communities and health facilities. All literate respondents provided written informed consent while illiterate respondents thumb-printed the informed consent form before participating in the study.

For the purposes of clarity this RCT was not a clinical trial because randomization into control and intervention groups was not done at the human subjects’ levels but at health facilities level. Health staff who by chance worked in intervention or control facilities were randomly sampled and interviewed. Similarly, household heads who by chance lived around the catchment area of intervention or control facilities were randomly interviewed. This study design did not demand a trial registration according to the ethical review protocols of the Ghana Health Service Ethical Review Committee. The health facilities were randomly assigned based on parameters such as outpatient and inpatient attendance, accreditation grade score, ownership and location (rural or urban).

### Statistical analysis

The data sets were analyzed with *Stata* statistical software (version 12.0) after data cleaning and coding to anonymize responses. To ensure internal validity, all questions were informed by research objectives and reviewed literature. The household and health provider data sets were merged into a single data set to enable comparison and cross tabulation of variables of interest.

Wilcoxon-Mann Whitney test was used to test the null hypothesis that perceptions of clients and health staff on the 12 service quality dimensions are not significantly different. Summary statistics (mean) were used to ascertain the average responses of staff and clients on the Likert scales [6,24] while descriptive statistics were performed on socio-demographic characteristics of clients and health staff.

Iterated principal factor (ipf) analysis was used categorize the 12 perceived/non-technical quality care dimensions into three main factors namely: “Complaint lodging, handling and feedback”; “Respect, compassion and supportiveness of staff” and “Adequacy of information provision, staff and services”. Because the outcome variable of interest is in ordinal scale, ordered logistic regression analysis was performed to ascertain whether or not patient safety and risk status (technical quality) significantly predicts client and health staff-perceived quality care. The outcome variables were measured by computing the average perception for a health facility by staff and clients based on the 12 quality care proxies. Control variables included in the regression models were respondents’ age, gender, marital status, religion, level of education, income levels (households only), and professional category (health staff only). Health facility ownership, rural-urban location and region were also controlled for in the regression model.

Multi-collinearity diagnostics were conducted on all explanatory variables of interest prior to their inclusion in the regression model and none had a variance inflation factor (VIF) above 10.0 [32]. The Likert scale responses used to fit the ordered logistic regression model fulfilled the proportional odds assumption following the Brant test [33]. Marginal effects of the explanatory variables in the regression model were also computed. Computation of marginal effects is one way to measure the effects of independent variables on a dependent variable. The marginal effect of an independent variable measures its impact of change on the expected change in the dependent variable, especially when the change in the independent variable is infinitely small or merely marginal [34].

## Results

### Socio-demographic characteristics of clients and health staff

Out of the 1,920 household questionnaires administered, a total of 1,903 household heads completely responded, representing a return rate of 99%. Out of the 333 health staff questionnaires administered, 324 were retrieved with complete responses representing a 97% return rate. The average number of household heads interviewed within the catchment area of a health facility was 30 and the average number of staff respondents per health facility was 5.

The average age for interviewed household heads was 45 years (SD = 15); 53% of them were married and 64% were males. A little over 50% of household heads had a maximum of basic education and 37% of them were insured; urban dwelling household heads constituted 50% of respondents, and 89% mentioned Christianity as their religion. At the health staff level, the average age of respondents was 39 years (SD = 14); 43% were married and 33% were males; 63% of the health staff had at least basic education qualification. Majority of health staff (72%) were insured and 44% of them worked in urban areas; Christianity was mentioned by 96% of the staff as their religion (see Table 1).

**Table 1:** Socio-demographic characteristics of clients and health staff.

Characteristics	Summary Statistics		
	Clients (n = 1,903)	Health Staff (n = 324)	p-value
Age (Mean, SD)	45 (15)	39 (14)	0.0000^b^
	**%**	**%**	
Males	64%	33%	0.077
Educational qualification			
Basic	54%	14%	0.001*
Secondary	15%	23%	
Tertiary	17%	63%	
Uneducated	14%	0%	
Clinical health staff^++^	NA	84%	NA
Married respondents	53%	43%	0.822
Employed respondents	86%	100%	0.537
Christian respondents	89%	96%	0.064
Location			
Rural	50%	56%	0.000**
Urban	50%	44%	
Region			
Greater Accra	50%	55%	0.000**
Western	50%	45%	
Currently insured^a^	37%	72%	0.716
Currently uninsured	53%	28%	

Source: WOTRO-COHEiSION Project Household and Health Facility Surveys (March, 2012)

^a^These include those insured with the NHIS and other forms of private health insurance schemes

^++^Clinical staff includes medical staff who have direct contact with patients (e.g. medical doctors, nursing personnel, pharmacy personnel, medical assistants and laboratory personnel. Non-clinical staffs are the support staff who do not have direct contact with patients (e.g. administrators, accountants, laborers and receptionists).

**Legend:** NA = Not applicable.

^b^t-test statistically significant (p<0.0001)

Pearson Chi-square test statistically significant

*p<0.05

**p<0.001

Clinical staff (n = 272) dominated the sample of health staff, comprising of medical doctors (n = 12), medical assistants (n = 13), midwives (n = 45), nursing staff (n = 138), pharmacy staff (n = 36) and laboratory staff (n = 28). Non-clinical staff (n = 52) comprised of accounting staff (n = 2), an administrator, NHIS contact persons/claims officers (n = 43), receptionists (n = 2), medical records officers (n = 3) and a secretary.

### Differences in client and staff perceptions of quality of health services

The results showed significant perception differences on the selected healthcare quality indicators by clients and health staff. Overall, health staff perceived many of the quality care indicators to be satisfactory in the NHIS-accredited health facilities, in contrast to clients (p<0.0001). Wider staff-client perception gaps/differences were observed in the areas of “satisfaction with health services provision by health staff” (staff mean = 3.62; client mean = 1.95, p<0.0001); “information to clients by health facility” (staff mean = 2.07; client mean = 3.68, p<0.0001) (see Table 2).

**Table 2:** Differences in client and health staff perceptions of healthcare quality.

Quality care proxies	Mean Ratings			Wilcoxon test	
	Health staff	Client	Mean Diff.	z-stat.	p-value
**Complaint lodging, handling and feedback**	**Mean** ^b^	**Mean**			
Avenues for complaint in health facility	3.57	2.49	1.09	33.5	0.0000^a^
Satisfaction with place/desk for lodging complaints	3.66	2.68	0.98	35.3	0.0000^a^
Process of lodging complaint at the health facility	3.61	2.73	0.88	33.0	0.0000^a^
**Respect, compassion and supportiveness of health staff**	**Mean**	**Mean**			
Compassion and supportiveness of health personnel	3.31	1.90	1.41	30.4	0.0000^a^
Respectfulness of doctors/medical assistants/nurses	3.24	1.91	1.34	-37.8	0.0000^a^
Equal treatment for insured and uninsured patients	1.24	2.48	-1.24	16.4	0.0000^a^
**Adequacy of information provision, staff and services**	**Mean**	**Mean**			
Adequacy of consulting rooms and medical equipment	2.74	2.32	0.41	24.5	0.0000^a^
Access to all prescribed drugs from the facility	3.00	2.38	0.62	36.7	0.0000^a^
Satisfaction with health services provided by health staff	3.62	1.95	1.66	37.4	0.0000^a^
Information provided by the health facility	2.07	3.68	-1.61	22.3	0.0000^a^
Sufficiency of good Docs./Med. Assistants/Nurses	2.63	2.11	0.52	34.3	0.0000^a^
Overall waiting time at the health facility	3.24	2.14	1.11	37.3	0.0000^a^

**Source:** WOTRO-COHEiSION Project Household and Health Facility Surveys (March, 2012)

^a^Wilcoxon-Mann Whitney test statistically significant (p<0.0001)

^b^Mean values are based on average rated satisfaction rankings of clients and staff per health facility. High values suggest better satisfaction with particular quality care component while lower values depict least satisfaction with quality of health services. Comparison for differences between staff and clients responses was done using the Wilcoxon-Mann Whitney test.

Other perceptions gaps were on “compassion and supportiveness of health personnel” (staff mean = 3.31; client mean = 1.90, p<0.0001); “respectfulness of doctors/medical assistants/nurses” (staff mean = 3.24; client mean = 1.91, p<0.0001); “equal treatment for insured and uninsured patients” (staff mean = 1.24; client mean = 2.48, p<0.0001), and “overall waiting time at the health facility” (staff mean = 3.24; client mean = 2.14, p<0.0001). While staff appeared to express satisfaction with many of these quality care markers, clients seemed to be disappointed (see Table 2).

### Technical quality care in sampled health facilities

All 64 sampled health facilities were assessed using the *SafeCare Essentials* tool, representing 100% participation. The results indicate that technical healthcare quality in the 64 sampled health facilities was generally low with an overall average score of 1.07 (SD = 0.22) out of the ideal score of 3.00 (see Table 3). Majority of the health facilities scored particularly low marks in the areas of “clinical outcomes monitoring” (mean = 0.08, SD = 0.27), “availability and use of clinical guidelines” (mean = 0.23, SD = 0.56), “correct identification of patients” (mean = 0.19, SD = 0.59), “communication among healthcare providers (mean = 0.39, SD = 0.73)”, “availability of policies and procedures for high risk patients (mean = 0.50, SD = 0.82)”, “presence of fire safety program” (mean = 0.47, SD = 0.50), and “appropriateness of surgical services (mean = 0.53, SD = 0.69)”.

**Table 3:** Average score per technical quality care proxy (n = 64).

Risk assessment areas (technical quality proxies)	Statistics			
Leadership process and accountability	Mean^f^	Std. Dev.	Min.	Max.
1.Leadership and accountability responsibilities are defined	1.91	0.39	0	3
2.Leadership for quality and patient safety	1.67	0.47	1	2
3.Day to day planning is collaborative	1.42	0.64	0	2
4.Clinical and managerial contracts are effectively managed	1.53	0.67	0	2
5.Compliance with all laws and regulations related to the clinic	0.88	0.63	0	2
6.Clear commitment to patient and family rights	1.89	0.31	1	2
7.Policies and procedures for high-risk procedures and patients	0.50	0.82	0	3
**Competent and capable workforce**				
8.All staff have personal files and job descriptions	1.70	0.49	1	3
9.The credentials for physicians are reviewed	1.28	0.45	1	2
10.The credentials for nurses and other health professionals are reviewed	1.39	0.61	0	2
11.Staff members are oriented to their jobs	0.94	0.43	0	2
12.Patient care staff are trained in resuscitative techniques	1.20	0.41	1	2
13.Staff are educated on infection prevention and control	1.23	0.43	1	2
14.Communication among those caring for the patients	0.39	0.73	0	2
**Safe environment for staff and patients**				
15.Regular maintenance for buildings	0.81	0.50	0	2
16.Control of hazardous materials	0.91	0.39	0	2
17.There is a fire safety programme	0.47	0.50	0	1
18.Biomedical equipment is maintained in a safe condition	1.52	0.62	0	2
19.Stable water and electricity sources are available	0.80	0.69	0	2
20.Reduction of health care-associated infections through proper hand hygiene	1.00	0.78	0	3
21.Barrier techniques are used	1.61	0.52	0	2
22.Proper disposal of sharps and needles	1.30	0.52	0	2
23.Proper disposal of infectious waste	1.91	0.40	0	2
24.Appropriate sterilization and cleaning procedures are used	0.73	0.57	0	2
**Clinical care of patients**				
25.Correct patient identification	0.19	0.59	0	3
26.Patient education about high risk procedures and informed consent	1.41	0.68	0	2
27.Medical and nursing assessments for all patients	0.61	0.70	0	2
28.Laboratory services are available and reliable	1.53	0.50	1	2
29.Diagnostic imaging services available, safe and reliable	1.39	0.52	0	2
30.Anesthesia and sedation are used appropriately	1.22	0.52	0	2
31.Surgical services are appropriate to patients needs	0.53	0.69	0	2
32.Medication use is safely managed	1.06	0.66	0	2
33.Patients are educated to participate in their care	1.06	0.43	0	2
34.Care that is planned and provided is written down in a patient record	1.41	0.64	0	3
**Improvement of quality and safety**				
35.There is a process for collecting and reviewing events that are unexpected and/potentially harmful to patients	1.05	0.33	0	2
36.High-risk processes are high-risk patients are monitored	1.19	0.43	0	2
37.Patient experience is monitored	1.09	0.46	0	2
38.There is a complaint process	1.02	0.42	0	2
39.Clinical guidelines and pathways are available and used	0.23	0.56	0	2
40.Staff understand how to improve processes	1.00	0.71	0	2
41.Clinical outcomes are monitored	0.08	0.27	0	1
**Overall average technical quality care** ^e^	**1.07**	**0.22**	**0.63**	**1.58**

**Source:** WOTRO-COHEiSION Project Household and Health Facility Surveys (March, 2012)

^e^Overall average technical quality care score computed by summing quality scores of all 64 facilities divided by the 41 quality care criteria.

^f^Mean scores depict the levels of effort demonstrated by health facilities per each risk area from 0–3 where high values suggest better performance towards patient safety and risk reduction and vice versa. Zero (0) is scored when the desired quality improvement activity in a clinic is absent or there is mostly *ad hoc* activity related to risk reduction. One (1) is scored when the structure of more uniform risk-reduction activity begins to emerge in a clinic. Two (2) is scored when there are processes in place for consistent and effective risk-reduction. Three (3) is scored when there are data to confirm successful risk-reduction strategies and continuous improvement.

Areas where most health facilities demonstrated comparatively better performance towards technical quality care improvement were: “leadership and accountability responsibilities” (mean = 1.91, SD = 0.39), “commitment to patients and family rights (mean = 0.89, SD = 0.31)”, and “proper disposal of infectious waste (mean = 0.91, SD = 0.40)” (see Table 3).

### Association between perceived and technical quality care in health facilities

Results of a Spearman’s correlation test (Table 4) showed that client perception of healthcare quality correlates negatively with technical quality care proxies (coef. = -0.0991, p<0.0001). In contrast, a strong positive correlation was observed between staff perception of healthcare quality and technical quality (coef. = 0.4600, p<0.0001). Likewise, client-perceived quality positively correlated with staff-perceived quality (coef. = 0.1054; p<0.0001) (see Table 4).

**Table 4:** Association between perceived and technical quality care.

Quality dimensions	Client-perceived quality^d^	Staff-perceived quality^e^	Technical quality
Client-perceived quality	1.0000		
Staff-perceived quality	0.1054**	1.0000	
Technical quality	-0.0991**	0.4600**	1.0000

^d,e^Staff and client perceived quality care were measured by computing the average perception for health facility by staff and clients using the 12 quality care proxies presented in Table 2.

**Spearman correlation coefficient statistically significant (p<0.0001)

### Factors associated with client and staff perception of healthcare quality

Ordered logistic regression results further confirmed that technical quality care in health facilities negatively correlated with client perception of service quality but positively correlated with staff-perceived quality care (p<0.05) (see Table 5). The results show that for one unit increase in technical quality score, we expect a 0.018 decrease in the log odds of client perceiving quality care as very satisfactory, holding other variables constant (p<0.05). In the case of health care providers, a unit increase in technical quality score is expected to increase the log odds of staff perceiving quality care as very satisfactory by 0.11, holding other variables constant (p<0.0001).

**Table 5:** Bivariate analysis of predictors of client and staff perceived quality care with marginal effects.

**Model 1**	**Dependent variable: Overall client-perceived quality**			
**Independent variables**	**Coef.**	**Std. Err**	**Marginal Effect** ^**+**^	**(95%Conf. Int.)**
Technical quality	-0.018*	0.005	0.0002	-0.028 -0.007
Age (mean = 45 years)	-0.006*	0.003	0.0000	-0.011 -0.000
Females	1.0	1.0	1.0	1.0
Males	0.014	0.097	-0.0002	-0.176 0.204
Not married	1.0	1.0	1.0	1.0
Married	-0.138	0.092	-0.0019	-0.317 0.042
Other religions	1.0	1.0	1.0	1.0
Christian religion	-0.141	0.127	0.0020	-0.391 0.109
Educated	1.0	1.0	1.0	1.0
No formal education	-0.056	0.125	0.0008	-0.301 0.189
Public facility	1.0	1.0	1.0	1.0
Private facility	-0.353**	0.100	0.0049	-0.549 -0.158
Urban location	1.0	1.0	1.0	1.0
Rural location	0.684**	0.980	-0.0095	0.492 0.876
Wealth quintile 1	1.0	1.0	1.0	1.0
Wealth quintile 2	0.420**	0.118	-0.0078	0.188 0.651
Wealth quintile 3	0.496**	0.127	-0.0089	0.247 0.746
Wealth quintile 4	0.980**	0.130	-0.0143	0.724 1.236
Wealth quintile 5	0.915**	0.134	-0.0137	0.652 1.177
**Obs.**	**1,903**			
**Pseudo R2**	**0.0105**			
**Log Likelihood**	**-7496.86**			
**Prob > chi2**	**0.0000**			
**Model 2**	**Dependent variable: Staff-perceived quality**			
**Independent variables**	**Coef.**	**Std. Err**	**Marginal Effect** ^+^	**(95%Conf. Int.)**
Technical quality	0.11**	0.013	-0.0002	0.085 0.137
Age (mean = 39 years)	-0.00	0.008	-0.0000	-0.016 0.015
Females	1.0	1.0	1.0	1.0
Males	0.19	0.222	-0.0005	-0.248 0.624
Not married	1.0	1.0	1.0	1.0
Married health staff	-0.29	0.224	-0.3706	-0.725 0.151
Other religions	1.0	1.0	1.0	1.0
Christian religion	-0.30	0.499	-0.0023	-1.278 0.677
Other qualifications	1.0	1.0	1.0	1.0
Tertiary education	0.34	0.209	-0.0002	-0.071 0.748
Clinical staff	1.0	1.0	1.0	1.0
Non-clinical staff	-0.22	0.285	0.0006	-0.776 0.340
Urban location	1.0	1.0	1.0	1.0
Rural location	-0.97**	0.225	0.0004	-1.412 -0.529
Public health facility	1.0	1.0	1.0	1.0
Private health facility	0.57*	0.226	-0.0025	0.123 1.010
**Obs.**	**324**			
**Pseudo R2**	**0.0276**			
**Log Likelihood**	**-938.32**			
**Prob > chi2**	**0.0000**			

**Source:** WOTRO-COHEiSION Project Household and Health Facility Surveys (March, 2012)

*p<0.05

**p<0.0001

^+^Conditional marginal effects (Model CE: OIM): Marginal effects represent the in probability when the respective predictor/independent variables increase by one unit (i.e. 0 to 1 for binary variables and instantaneous change for continuous variables)

The change in probability for one instant change in technical quality score and clients’ age is almost 0.0 percentage point (p<0.05). The change in probability for public facility relative to private was 0.49 percentage point while that for rural against urban was 0.095 percentage point (p<0.05). In terms of wealth quintiles of clients, the change in probability for one unit increase in wealth appeared to reduce the marginal percentage points on clients responses (see Table 5) (p<0.05).

Analysis of the staff data showed that the change in probability for one unit increase in technical quality is approximately 0 percentage points while that for rural against urban location of staff was 0.04 percentage point (p<0.05). Clients who were located in rural areas and found in relatively lower wealth quintiles appeared to have better perspectives of healthcare quality than those located in urban areas and higher wealth quintiles (p<0.0001). Increasing client age did not seem to favour perceptions on healthcare quality (p<0.05). Health staff working in rural clinics/health centres also appeared to have negative perspectives of healthcare quality than their counterparts in urban health facilities (p<0.0001).

## Discussion

The study found that health staff’s perception of service quality correlates positively with technical quality. However, clients’ perceptions of service quality were negatively associated with technical quality in sampled health facilities. Staff and clients were found to have different perceptions of what constitutes healthcare quality. Overall, health staff perceived the quality of services they render to clients as satisfactory contrary to perceptions of clients who perceived the quality of services to be dissatisfactory. The low patient satisfaction levels with health service quality is consistent with findings of previous patient satisfaction surveys on Ghana [5,9,14,15,35,36].

These findings suggest there is room for improvement in quality of health service delivery, particularly from the client’s perspective. Intensifying efforts towards meeting expectations of clients while maintaining technical quality requirements will likely lead to higher client trust and confidence in service providers which is a good recipe for higher health insurance uptake, retention and utilization of safer healthcare services. The differences in perceptions of clients and staff could be attributed to a number of factors which include respondents’ understanding of the healthcare quality issues at stake. The relatively higher satisfaction ratings by health staff on many of the quality healthcare proxies could be attributed to tendency of health staff to give more favorable answers to portray “a good name” for their facilities or perhaps health staff were complacent of their efforts towards quality service delivery.

Information asymmetry between the health staff and clients also possibly explain the differences in perceptions on service quality. For instance, even though complaint systems might exist in health facilities if clients are not adequately informed on how to use them, client perceptions will remain low. Parasuraman et al [3] described this missing link as a quality care gap between clients’ expectations and perceptions of health providers on what clients expect. Effective bottom-up communication system between clients and service providers could help bridge this gap.

Routine community engagement sessions involving staff and clients on the components of healthcare quality could help improve the staff-client perception differences. These platforms will help educate participants on their rights and responsibilities and offer health providers the opportunity to improve on client-perceived quality care gaps. Clients should also be educated on the dynamics of healthcare delivery and the need for realistic expectations/demands cognizant of the available human and material resource capacity of health facilities.

Unbalanced commitments towards technical and perceived quality care improvement could be another reason for the negative association between technical and client-perceived quality care dimensions. Healthcare facilities which do not recognize clients have concerns with human relations of staff could lead them to perpetually render services that do not satisfy clients’ needs even though adherence to professional practices (technical quality) might be adequate. This gap could be addressed by stepping up client-centered care and community engagement interventions in the service delivery process. De Man et al [24] made similar proposal when they found that perspectives of staff and clients differed significantly on many quality care markers.

The technical quality assessment findings show low performance of sampled health facilities on many of the technical quality care criteria. The results showed that none of the mean scores attained by the health facilities were up to the 3.0 ideal score. This implies majority of the health facilities did not have uniform processes in place for consistent and effective patient safety. The results also suggest that many of the health facilities did not have data to confirm successful risk-reduction strategies and continuous improvement. All in all, the low mean scores depict potentially widespread *ad hoc* processes and activities related to risk reduction and patient safety (technical quality care).

These findings underscore the need for the National Health Insurance Authority (NHIA) to intensify routine post accreditation monitoring system that integrates non-technical quality care indicators into the mainstream monitoring tools to help promote client-centered quality care improvement while maintaining medical technical quality care standards. This approach could help enhance client trust and confidence in NHIS-accredited health facilities and contribute towards sustaining the NHIS.

The negative correlation between client-perceived quality care and technical quality in health facilities imply that improvement in technical quality *per se* will not necessarily correspond with increased client satisfaction with quality of health services. Robyn et al [23] made similar observations in a study in Burkina Faso where highly rated client satisfaction scores correlated negatively with adherence to technical quality care practices. Balanced commitment to both perceived and technical components of healthcare quality thus appear to be a better strategy towards wholistic healthcare quality improvement.

Besides the above posits, perhaps the negative association between technical and client-perceived quality care is due to the fact that the *SafeCare Essentials* tool by design has no informative value on client experiences and perception of service quality since it was mainly developed to measure technical components of healthcare. The tool does not take into account client-perceived quality care.

Even though intuitively one would expect that high efforts towards technical quality care translate into higher client-perceived quality, it is not always the case because of information asymmetry. For instance, some clients will likely describe good quality care to be prescription of large quantities of drugs and injections per outpatient visit but this would constitute irrational use of drugs or polypharmacy in medical practice. Moreover, a health staff may be perceived as unfriendly and/or disrespectful towards patients but professionally more competent than colleagues perceived to be friendly or respectful. Health illiteracy on the part of clients especially in many developing countries potentially misinforms clients in their interpretation of what constitutes good healthcare quality [37].

In sum, these findings highlight the need for health managers and policy makers to balance efforts towards technical quality improvement with functional quality dimensions such as attitudes of staff, timeliness of care and client support systems which clients perceive as important indicators of quality healthcare. This balanced approach can be achieved by incorporating functional quality dimensions into mainstream official quality monitoring and evaluation frameworks.

While acknowledging the importance of technical quality care standards, there is also the tendency for it to be over emphasized by health managers and policy makers to the neglect of non-technical quality care dimensions which do not often take much resources and efforts to improve. Intensified patient education, engagement and patient-friendly quality improvement interventions could help bridge these quality care gaps.

## Limitations

The authors acknowledge some limitations associated with this study. First, the study was conducted in two (2) out of ten (10) regions in Ghana, thus the sample size might not to be representative of the Ghanaian population. Respondents’ experiences of service quality could differ significantly in other regions of Ghana. Moreover, the outlier districts (in terms of remoteness and the PCA criteria), as well as outlier health facilities (in terms of accreditation scores and other PCA criteria) had less probability of being selected. In view of this limitation in sampling, the results could be influenced by the cadre of districts and health facilities sampled.

Secondly, only primary healthcare facilities (mostly located in rural areas) were sampled for the study implying that the findings might not reflect conditions in higher level facilities often located in better endowed urban areas.

Finally, the *SafeCare Essentials* criteria applied in this study were used as proxies of technical quality care. Detailed technical quality care assessment was not done due to limited time and resources available to the researchers. Nonetheless, the tool remains relevant to the Ghanaian healthcare system because it gives a snapshot of the healthcare quality challenges confronting health facilities. Moreover, implementation of the tool in Ghana and other African countries such as Tanzania, Nigeria, Mozambique, Namibia and Kenya gives credence to its relevance and appropriateness for this study. In light this, the tool is proposed to the National Health Insurance Authority (NHIA) for possible adoption as an NHIA rapid pre-accreditation tool for public and private facilities to help enhance performance of health facilities during accreditation.

## Policy Recommendations

Based on the findings of the study the following recommendations are proposed:

1. The Ghana Health Service (GHS)/Ministry of Health (MoH) should initiate discussions on a possible staff appraisal system that incorporates feedback on staff performance from clients or organized community-based groups/associations. This could help make health staff more accountable to clients and promote client-centered quality care delivery. The feasibility of this initiative should however be piloted and mindful of the mobile nature of clients and staff.
2. Communities should be empowered through active engagement in routine assessment of the quality of services rendered by health facilities and reward systems given to facilities that are perceived by the community to be client-centered. This could encourage healthy competition among facilities and promote a balanced approach to quality improvement.
3. The NHIA should decentralize and effectively monitor its post accreditation monitoring for NHIS-accredited health facilities to ensure quality standards are maintained after accreditation. District level NHIA offices should be well resourced to undertake these monitoring activities more frequently and effectively.
4. Finally, the NHIA should initiate policy dialogues and stakeholder consultations on possibly integrating non-technical quality care dimensions into its post accreditation monitoring framework health facilities. This will help motivate facilities to prioritize client-centered quality services.

## Conclusion

Quality of healthcare as perceived by clients and per the *SafeCare Essentials* assessment is low in majority of the sampled NHIS-accredited health facilities in Ghana. Contrary to clients, it appeared health staff perceive the quality care situation to be good, evident in their higher satisfaction ratings on quality care markers. These differences are indicative of a possible communication gap and information asymmetry between clients and service providers. There is the need for quality improvement efforts that prioritize client-centered quality, especially in primary healthcare facilities which constitute over 70% of the over 3,000 NHIS-accredited health facilities in Ghana. These cadre of health facilities provide basic primary healthcare services which is critical to sustain the gatekeeper system under the NHIS.

Client-centered approach will help improve the existing information asymmetry between clients and service providers on what constitutes quality care and mitigate unrealistic expectations from clients. Clients’ measure of quality healthcare usually hinges on interpersonal and non-technical quality indicators that health providers might overlook. While acknowledging the importance of medical technical quality in health service delivery, balancing it with client-perceived quality will prove beneficial towards enhancing client confidence and trust in the healthcare system which is essential for a viable health insurance system in Ghana and Africa at large.

## Abbreviations

GAR: Greater Accra Region
GHS: Ghana Health Service
MoH: Ministry of Health
NHIA: National Health Insurance Authority
NHIS: National Health Insurance Scheme
WR: Western Region
QA: Quality Assurance
MDGs: Millennium Development Goals
WHO: World Health Organization
ICD: Institutional Care Division
IoM: Institute of Medicine
EAs: Enumeration Areas
RCT: Randomized Control Trial
LIMCs: Low and Middle Income Countries
